# Kinetics of Ion-Capturing/Ion-Releasing Processes in Liquid Crystal Devices Utilizing Contaminated Nanoparticles and Alignment Films

**DOI:** 10.3390/nano8020059

**Published:** 2018-01-23

**Authors:** Yuriy Garbovskiy

**Affiliations:** UCCS BioFrontiers Center and Department of Physics, University of Colorado Colorado Springs, Colorado Springs, CO 80918, USA; ygarbovs@uccs.edu or ygarbovskiy@gmail.com; Tel.: +1-719-255-3123

**Keywords:** liquid crystals, ions, contaminated nanoparticles, kinetics, ion-capturing films, ion trapping, adsorption/desorption

## Abstract

Various types of nanomaterials and alignment layers are considered major components of the next generation of advanced liquid crystal devices. While the steady-state properties of ion-capturing/ion-releasing processes in liquid crystals doped with nanoparticles and sandwiched between alignment films are relatively well understood, the kinetics of these phenomena remains practically unexplored. In this paper, the time dependence of ion-capturing/ion-releasing processes in liquid crystal cells utilizing contaminated nanoparticles and alignment layers is analyzed. The ionic contamination of both nanodopants and alignment films governs the switching between ion-capturing and ion-releasing regimes. The time dependence (both monotonous and non-monotonous) of these processes is characterized by time constants originated from the presence of nanoparticles and films, respectively. These time constants depend on the ion adsorption/ion desorption parameters and can be tuned by changing the concentration of nanoparticles, their size, and the cell thickness.

## 1. Introduction

Ions in liquid crystals can affect the performance of devices utilizing these materials in different ways. In the majority of cases, liquid crystal devices (displays (LCD), tunable wave plates and variable retarders, filters, and lenses) are driven by electric fields reorienting liquid crystal molecules and changing the properties of the device [1,2]. For this type of applications, ions in liquid crystals are very undesirable objects since they can lead to many negative side effects (image sticking, image flickering, reduced voltage holding ratio, and slow response) thus compromising an overall performance of liquid crystal devices [2,3]. That is why the development of new methods to purify liquid crystals from ions is of utmost importance to the LCD industry. There are also an increasing number of applications relying on ions in liquid crystals such as liquid crystal shutters and optical switches utilizing light scattering effects [4,5,6].

The dispersion of nanomaterials in liquid crystals has recently emerged as a promising way to control the concentration of mobile ions in liquid crystals ([7] and references therein). The most widely used nanomaterials include carbon-based nano-objects [7,8], dielectric [7,9], semiconductor [7,10], magnetic [7,11], metal [7,12], and ferroelectric [7,13,14] nanoparticles. Numerous studies revealed the complex behavior of nanoparticles in liquid crystals and the possibility of several regimes, namely the ion-capturing regime (the purification of liquid crystals from ions), ion-releasing regime (the contamination of liquid crystals with ions), and no change in the concentration of ions [7,15,16]. The ionic contamination of nanomaterials is considered a major factor determining the type of the regime achieved in experiments [15,16,17,18,19,20,21,22,23,24].

So far, the effects of nanomaterials on the concentration of mobile ions in liquid crystals were studied under equilibrium conditions mostly. There are very limited number of papers reporting the time dependence of the concentration of ions n(t) in liquid crystals doped with nanomaterials [25,26]. Moreover, the kinetics of ion-capturing/ion-releasing regimes in liquid crystals doped with contaminated nanoparticles was not discussed at all. Alignment layers constitute a major component of practically any liquid crystal device [1,2,27]. Therefore, it is also very important to consider the combined effect of both nanoparticles and alignment layers on the time dependence n(t). The steady-state ion-capturing properties of several types of alignment layers including polymer-based films [28,29,30], SiO*_x_*-based films [31,32,33], and films made of graphene [34] have been reported. While in the case of liquid crystal cells utilizing polyimide alignment layers the kinetics of ion adsorption/ion desorption processes causing the ion-capturing effect was studied in several publications [35,36,37,38,39], there are no publications focused on the kinetics of ion-capturing/ion-releasing processes in liquid crystals sandwiched between contaminated alignment layers. In addition, the combined effect of contaminated nanoparticles and alignment layers on the time dependence of ion-capturing/ion-releasing regimes is also not discussed in existing literature. This paper is aimed at analyzing the kinetics of these regimes in liquid crystal cells utilizing nanomaterials and alignment layers contaminated with ions.

## 2. Theoretical Model and Results

### 2.1. Contaminated Nanoparticles in Liquid Crystals

Consider liquid crystals doped with nanoparticles. To simplify the discussion, both liquid crystals and contaminated nanoparticles are characterized by the same type of fully ionized ionic species. In this case, the ion adsorption/ion desorption processes change the concentration of mobile ions in liquid crystals according to the rate Equation (1):

(1) $$\frac{dn}{dt}=-k_{a}^{NP}n_{NP}A_{NP}\sigma_{S}^{NP}n\left(1-\Theta_{NP}\right)+k_{d}^{NP}n_{NP}A_{NP}\sigma_{S}^{NP}\Theta_{NP}$$

where n is the concentration of mobile ions, $A_{NP}$ is the surface area of a single nanoparticle; $n_{NP}$ is the volume concentration of nanoparticles; $\sigma_{S}^{NP}$ is the surface density of all adsorption sites on the surface of a single nanoparticle; $k_{a}^{NP}$ is the adsorption rate constant; and $k_{d}^{NP}$ is the desorption rate constant; $\Theta_{NP}$ is the fractional surface coverage of nanoparticles defined as $\Theta_{NP}=\frac{\sigma_{NP}}{\sigma_{S}^{NP}}$ ($\sigma_{NP}$ is the surface density of adsorption sites on the surface of nanoparticles occupied by ions). The first term of Equation (1) accounts for the adsorption of ions onto the surface of nanoparticles, and the second term describes the ion desorption from the surface of nanoparticles. In the steady-state regime (dndt=0) Equation (1) reduces to the Langmuir adsorption isotherm [40]. The discussion of the applicability and limitations of this approach to compute the concentration of mobile ions in liquid crystals can be found in recently published papers [41,42,43].

The conservation law of the total number of ions can be written as Equation (2):

(2) $$n_{0}+n_{NP}A_{NP}\sigma_{S}^{NP}\nu_{NP}=n+n_{NP}A_{NP}\sigma_{S}^{NP}\Theta_{NP}$$

where $n_{0}$ is the initial concentration of ions in liquid crystals, and $\nu_{NP}$ is the contamination factor of nanoparticles. The contamination factor of nanoparticles accounts for their possible ionic contamination [15]. It equals the fraction of the adsorption sites on the surface of nanoparticles occupied by ions-contaminants prior to dispersing them in liquid crystals [15].

The kinetics of ion-capturing/ion-releasing processes in liquid crystals doped with contaminated nanoparticles can be computed by solving Equations (1) and (2). These equations can be solved analytically [44,45,46]. The general analytical solution is very bulky and not easy to analyze [46]. However, in the majority of the reported experimental studies the observed fractional surface coverage is very low, $\Theta_{NP}<<1$ [16,21]. It allows for some simplifications. In the regime of relatively low surface coverage, an analytical solution can be written as Equation (3):

(3) $$n\approx \frac{n_{0}+n_{NP}A_{NP}\sigma_{S}^{NP}\nu_{NP}+n_{NP}A_{NP}\sigma_{S}^{NP}\left(K_{NP}n_{0}-\nu_{NP}\right)e^{-\left(k_{a}^{NP}n_{NP}A_{NP}\sigma_{S}^{NP}+k_{d}^{NP}\right)\;t}}{1+K_{NP}n_{NP}A_{NP}\sigma_{S}^{NP}}$$

In real systems, the values of physical parameters characterizing adsorption-desorption processes ($\sigma_{S}^{NP}$, KNP=kaNPkdNP) can vary within very broad limits (Table 1). Therefore, in the present study, their values were selected to reasonably represent existing materials.

**Figure 1:**
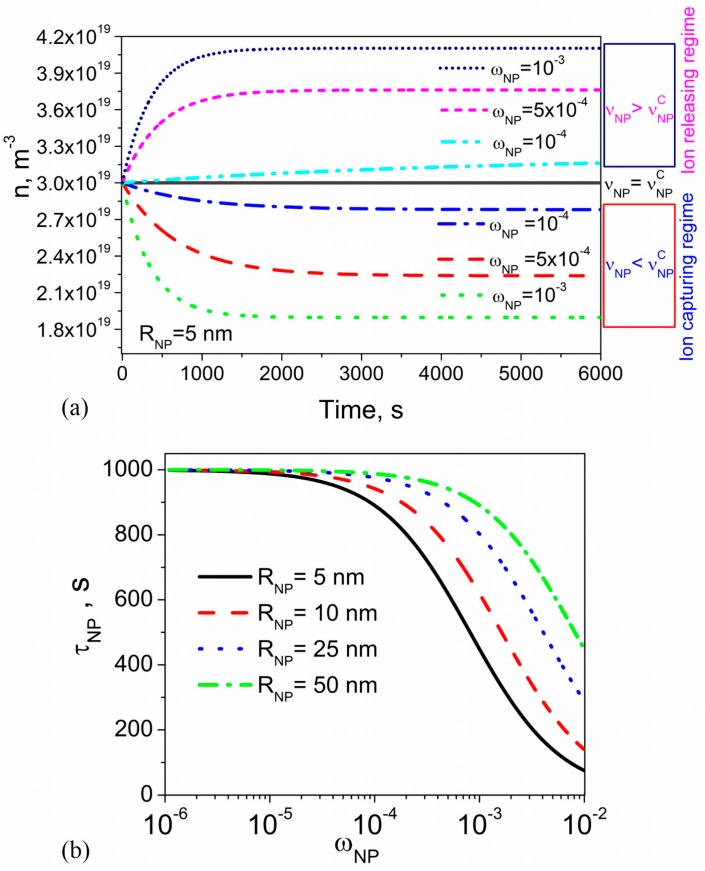
(**a**) The volume concentration of mobile ions n versus time calculated using different values of the weight concentration of nanoparticles $\omega_{NP}$ and their contamination factor $\nu_{NP}$ ($v_{NP}=10^{-4}$ (dotted, dashed, and dotted-dashed curves); $v_{NP}=3\times 10^{-4}$ (solid curve); $v_{NP}=5\times 10^{-4}$ (dashed-dotted-dotted, short-dashed, and short-dotted curves)). The radius of nanoparticles $R_{NP}$ is 5 nm; (**b**) The time constant $\tau_{NP}$ as a function of the weight concentration of nanoparticles $\omega_{NP}$ calculated at different values of the nanoparticle radius $R_{NP}$ ($R_{NP}=5\;\mathrm{nm}$ (solid curve); $R_{NP}=10\;\mathrm{nm}$ (dashed curve); $R_{NP}=25\;\mathrm{nm}$ (dotted curve); $R_{NP}=50\;\mathrm{nm}$ (dashed-dotted curve)). Other parameters used in simulations: $K_{NP}=10^{-23}\;m^{3}$, $k_{d}^{NP}=10^{-3}\;s^{-1}$, $\sigma_{S}^{NP}=0.8\times 10^{18}\;m^{-2}$, $n_{0}=3\times 10^{19}\;m^{-3}$, $\rho_{NP}/\rho_{LC}=3.9$.

An example of typical time dependence of the concentration of mobile ions in liquid crystals doped with contaminated nanoparticles is shown in Figure 1. As can be seen from Figure 1a, the use of contaminated nanoparticles results in the possibility of several regimes, namely the ion-capturing regime (dotted, dashed, and dashed-dotted curves), ion-releasing regime (dashed-dotted-dotted, short-dashed, and short-dotted curves), and no change regime (solid curve). The switching between these regimes is governed by the contamination level of nanoparticles: the ion-capturing regime is observed if $\nu_{NP}<\nu_{NP}^{C}$, the ion-releasing regime holds true if $\nu_{NP}>\nu_{NP}^{C}$, and no change regime is reached if $\nu_{NP}=\nu_{NP}^{C}$, where $\nu_{NP}^{C}$ is the critical contamination factor of nanoparticles defined as $\nu_{NP}^{C}=\frac{n_{0}K_{NP}}{1+n_{0}K_{NP}}\approx n_{0}K_{NP}$ where KNP=kaNPkdNP. Both ion-releasing and ion-capturing regimes are more pronounced at higher concentration of nanoparticles (Figure 1a). Figure 1a also indicates that the time needed to reach the steady state (dndt=0) depends on the concentration of nanoparticles and decreases at higher concentrations. 

The kinetics of ion-releasing/ion-capturing processes in liquid crystals doped with nanoparticles is characterized by the time constant $\tau_{NP}$ describing how rapidly the steady-state can be reached. This time constant can be defined using a standard definition: $n(\tau_{NP})-n_{0}=\left(1-1/e\right)\left(n_{\infty }-n_{0}\right)$, where $n_{0}=n(t=0)$ and $n_{\infty }=n(t\to \infty )$. According to Equation (3), in the regime of low surface coverage, $\Theta_{NP}<<1$, the time constant can be expressed as $\tau_{NP}=1/k_{d}^{NP}\left(K_{NP}n_{NP}A_{NP}\sigma_{S}^{NP}+1\right)$. Using the relationship between the volume and weight concentration of nanoparticles ($n_{NP}\approx \omega_{NP}\frac{\rho_{LC}}{\rho_{NP}}\frac{1}{V_{NP}}$, where $V_{NP}$ is the volume of a single nanoparticle, and $\rho_{LC}$ ($\rho_{NP}$) is the density of liquid crystals (nanoparticles)), the time constant can also be rewritten as $\tau_{NP}\approx 1/k_{d}^{NP}\left(3K_{NP}\sigma_{S}^{NP}\omega_{NP}\frac{\rho_{LC}}{\rho_{NP}R_{NP}}+1\right)$, where $R_{NP}$ is the radius of spherical nanoparticles. As can be seen, the time constant $\tau_{NP}$ depends on the adsorption-desorption parameters (kdNP,KNP=kaNPkdNP,σSNP), the concentration of nanoparticles $\omega_{NP}$, and their size $R_{NP}$. The dependence of the time constant on the weight concentration of nanoparticles calculated at four different values of their radius is shown in Figure 1b. An increase in the concentration of nanoparticles results in the monotonous decrease of the time constant (Figure 1b). At the same concentration of nanoparticles $\omega_{NP}$, the time constant $\tau_{NP}$ is shorter for smaller nanoparticles (Figure 1b). 

**Figure 2:**
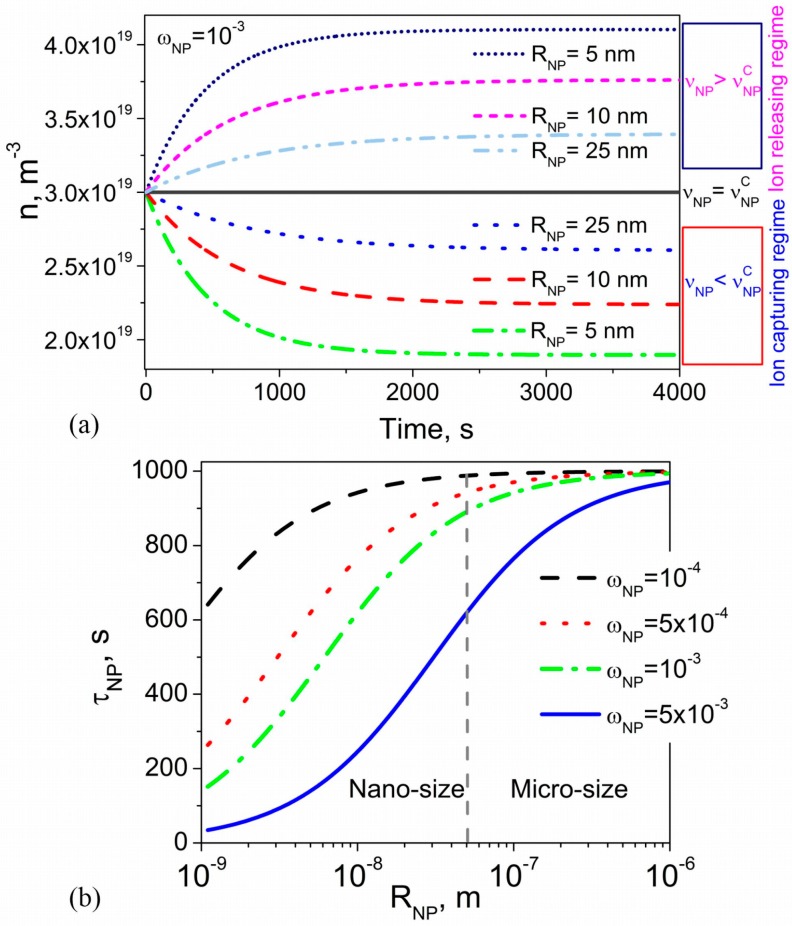
(**a**) The volume concentration of mobile ions n versus time calculated using different values of the nanoparticle radius $R_{NP}$ ($R_{NP}=5\;\mathrm{nm}$ (dotted-dashed and short-dotted curves); $R_{NP}=10\;\mathrm{nm}$ (dashed and short-dashed curves), $R_{NP}=25\;\mathrm{nm}$ (dotted and dashed-dotted-dotted curves) and their contamination factor $\nu_{NP}$ ($v_{NP}=10^{-4}$ (dotted, dashed, and dotted-dashed curves); $v_{NP}=3\times 10^{-4}$ (solid curve); $v_{NP}=5\times 10^{-4}$ (dashed-dotted-dotted, short-dashed, and short-dotted curves)). The weight concentration of nanoparticles $\omega_{NP}$ is 10^−3^; (**b**) The time constant $\tau_{NP}$ as a function of the radius of nanoparticles $R_{NP}$ calculated at different values of their weight concentration $\omega_{NP}$ ($\omega_{NP}=10^{-4}$ (dashed curve); $\omega_{NP}=5\times 10^{-4}$ (dotted curve); $\omega_{NP}=10^{-3}$ (dashed-dotted curve); $\omega_{NP}=5\times 10^{-3}$ (solid curve)). Other parameters used in simulations: $K_{NP}=10^{-23}\;m^{3}$, $k_{d}^{NP}=10^{-3}\;s^{-1}$, $\sigma_{S}^{NP}=0.8\times 10^{18}\;m^{-2}$, $n_{0}=3\times 10^{19}\;m^{-3}$, $\rho_{NP}/\rho_{LC}=3.9$.

The time dependence n(t) calculated using the fixed weight concentration of nanoparticles and different values of the nanoparticle radius is shown in Figure 2a. According to this figure, the time needed to reach steady-state depends on the size of nanoparticles. This time (the time constant) is shorter if smaller nanoparticles are used. The dependence of the time constant $\tau_{NP}$ on the radius of nanoparticles is shown in Figure 2b. As can be seen, $\tau_{NP}$ can be significantly reduced by utilizing smaller nanoparticles and by increasing their concentration. 

### 2.2. The Effects of Contaminated Alignment Layers

The kinetics shown in Figure 1 and Figure 2 was modelled ignoring interactions of ions with alignment layers of the liquid crystal cell. To account for possible effects associated with the adsorption of ions onto the surface of alignment layers, consider sandwich-like cell filled with *pristine* liquid crystals (without nanoparticles). In this case, the change in the concentration of mobile ions in liquid crystals through their adsorption onto the surface of alignment layers can be described by the following rate Equation (4):

(4) $$\frac{dn}{dt}=-k_{a}^{S}\frac{\sigma_{S}^{S}}{d}n\left(1-\Theta_{S}\right)+k_{d}^{S}\frac{\sigma_{S}^{S}}{d}\Theta_{S}$$

where n is the concentration of mobile ions; $\sigma_{S}^{S}$ is the surface density of all adsorption sites on the surface of alignment layers; d is the cell thickness; $k_{a}^{S}$ is the adsorption rate constant describing the ion adsorption onto the surface of alignment layers; and $k_{d}^{S}$ is the desorption rate constant; $\Theta_{S}$ is the fractional surface coverage of alignment layers. To compute the time dependence n(t), Equation (4) should be solved together with Equation (5) representing the conservation law of the total number of ions in the liquid crystal cell:

(5) $$n_{0}+\frac{\sigma_{S}^{S}}{d}\nu_{S}=n+\frac{\sigma_{S}^{S}}{d}\nu_{S}\Theta_{S}$$

where $\nu_{S}$ is the contamination factor of substrates (alignment layers). It equals the fraction of the adsorption sites on the surface of alignment layers occupied by ions-contaminants prior to filling an empty cell with liquid crystals [43,50]. Mathematically, Equations (1), (2), (4) and (5) are similar. In the regime of relatively small surface coverage, $\Theta_{S}<<1$, an analytical solution can be expressed by Equation (6):

(6) $$n\approx \frac{n_{0}+\frac{\sigma_{S}^{S}}{d}\nu_{S}+\frac{\sigma_{S}^{S}K_{S}}{d}\left(n_{0}-\frac{\nu_{S}}{K_{S}}\right)e^{-\left(k_{a}^{S}\frac{\sigma_{S}^{S}}{d}+k_{d}^{S}\right)t}}{1+\frac{\sigma_{S}^{S}K_{S}}{d}}$$

Typical values of physical parameters ($\sigma_{S}^{S}$, $k_{d}^{S}$, KS=kaSkdS) characterizing existing materials are compiled in Table 2.

**Figure 3:**
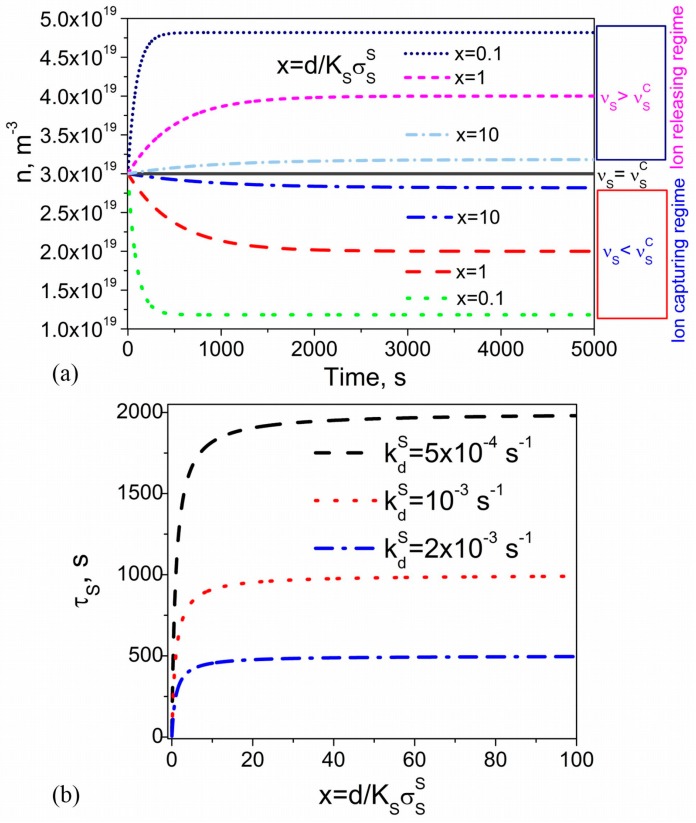
(**a**) The volume concentration of mobile ions n versus time calculated using different values of the dimensionless parameter x proportional to the cell thickness (x=dKSσSS). The ionic contamination of substrates is quantified by means of the contamination factor $\nu_{S}$ ($v_{S}=10^{-3}$ (dotted, dashed, and dotted-dashed curves); $v_{S}=3\times 10^{-3}$ (solid curve); $v_{S}=5\times 10^{-3}$ (dotted-dashed-dashed, short-dashed, and short-dotted curves)). Other parameters used in simulations: $K_{S}=10^{-22}\;m^{3}$, $k_{d}^{S}=10^{-3}\;s^{-1}$, $\sigma_{S}^{S}=10^{17}\;m^{-2}$, $n_{0}=3\times 10^{19}\;m^{-3}$; (**b**) The time constant $\tau_{S}$ as a function of the dimensionless parameter x calculated at different values of the desorption rate coefficient, $k_{d}^{S}$ ($k_{d}^{S}=5\times 10^{-4}\;s^{-1}$ (dashed curve); $k_{d}^{S}=10^{-3}\;s^{-1}$ (dotted curve); $k_{d}^{S}=2\times 10^{-3}\;s^{-1}$ (dashed-dotted curve)).

According to Figure 3a, the type of the observed regime is governed by the ionic contamination of substrates: the ion-capturing regime is reached if $\nu_{S}<\nu_{S}^{C}$ (dotted, dashed, and dashed-dotted curves), the ion-releasing regime takes place if $\nu_{S}>\nu_{S}^{C}$ (dotted-dashed-dashed, short-dashed, and short-dotted curves), and nothing happens if $\nu_{S}=\nu_{S}^{C}$ (solid curve). The time dependence shown in Figure 3a can also be characterized by the time constant $\tau_{S}$ defined as $n(\tau_{S})-n_{0}=\left(1-1/e\right)\left(n_{\infty }-n_{0}\right)$, where $n_{0}=n(t=0)$ and $n_{\infty }=n(t\to \infty )$. This time constant depends on the cell thickness. It decreases if the cell gap decreases (Figure 3a). According to Equation (6), in the regime of low surface coverage, $\Theta_{S}<<1$, it can be expressed as τS=1kdS(KSσSSd+1)=1kdS(1x+1). The dependence of the time constant $\tau_{S}$ on the cell thickness is shown in Figure 3b. As can be seen, the effects of the cell thickness on the time constant are strongly pronounced if relatively thin cells ($d<<K_{S}\sigma_{S}^{S}$) are used. These effects become negligible in the case of relatively thick cells ($d>>K_{S}\sigma_{S}^{S}$).

### 2.3. The Combined Effect of Contaminated Nanoparticles and Substrates

By combining Equations (1) and (4), we can write the generalized rate Equation (7) describing the combined effect of contaminated nanoparticles and alignment layers on the time dependence of the ion-capturing/ion-releasing regimes in liquid crystals:

(7) $$\frac{dn}{dt}=-k_{a}^{NP}n_{NP}A_{NP}\sigma_{S}^{NP}n\left(1-\Theta_{NP}\right)+k_{d}^{NP}n_{NP}A_{NP}\sigma_{S}^{NP}\Theta_{NP}-k_{a}^{S}\frac{\sigma_{S}^{S}}{d}n\left(1-\Theta_{S}\right)+k_{d}^{S}\frac{\sigma_{S}^{S}}{d}\Theta_{S}$$

In this case, the kinetics of ion-capturing/ion-releasing processes can be analyzed by solving Equation (7) along with the conservation law of the total number of ions written in more general form (8):

(8) $$n_{0}+n_{NP}A_{NP}\sigma_{S}^{NP}\nu_{NP}+\frac{\sigma_{S}^{S}}{d}\nu_{S}=n+n_{NP}A_{NP}\sigma_{S}^{NP}\Theta_{NP}+\frac{\sigma_{S}^{S}}{d}\Theta_{S}$$

In the regime of relatively low surface coverages, $\Theta_{NP}<<1$ and $\Theta_{S}<<1$, an analytical solution can be written as Equation (9):

(9) $$n\approx \frac{n_{0}+n_{NP}A_{NP}\sigma_{S}^{NP}\nu_{NP}+\frac{\sigma_{S}^{S}\nu_{S}}{d}+n_{NP}A_{NP}\sigma_{S}^{NP}K_{NP}\left(n_{0}-\frac{\nu_{NP}}{K_{NP}}\right)e^{-\left(k_{a}^{NP}n_{NP}A_{NP}\sigma_{S}^{NP}+k_{d}^{NP}\right)\;t}+\frac{\sigma_{S}^{S}K_{S}}{d}\left(n_{0}-\frac{\nu_{S}}{K_{S}}\right)e^{-\left(k_{a}^{S}\frac{\sigma_{S}^{S}}{d}+k_{d}^{S}\right)t}}{1+K_{NP}n_{NP}A_{NP}\sigma_{S}^{NP}+\frac{\sigma_{S}^{S}K_{S}}{d}}$$

**Figure 4:**
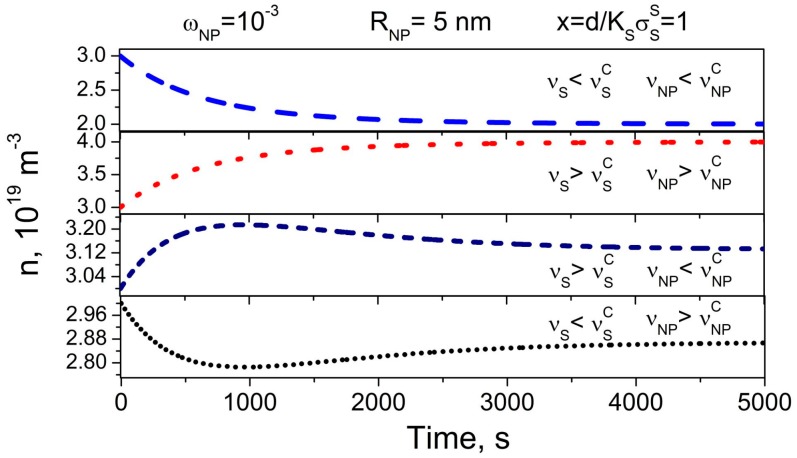
Time dependence of the volume concentration of mobile ions n in liquid crystals doped with contaminated nanoparticles and sandwiched between contaminated substrates. The radius of nanoparticles is 5 nm, and their weight concentration is 0.001. The dimensionless parameter x=1. The contamination factors of both nanoparticles and substrates are varied: dashed curve ($v_{S}=10^{-3}<\nu_{S}^{C}=3\times 10^{-3}$, $v_{NP}=2\times 10^{-4}<\nu_{NP}^{C}=3\times 10^{-4}$); dotted curve ($v_{S}=5\times 10^{-3}>\nu_{S}^{C}=3\times 10^{-3}$, $v_{NP}=4\times 10^{-4}>\nu_{NP}^{C}=3\times 10^{-4}$); short-dashed ($v_{S}=5\times 10^{-3}>\nu_{S}^{C}=3\times 10^{-3}$, $v_{NP}=2\times 10^{-4}<\nu_{NP}^{C}=3\times 10^{-4}$); short-dotted ($v_{S}=10^{-3}<\nu_{S}^{C}=3\times 10^{-3}$, $v_{NP}=4\times 10^{-4}>\nu_{NP}^{C}=3\times 10^{-4}$). Other parameters used in simulations: $K_{S}=10^{-22}\;m^{3}$, $K_{NP}=10^{-23}m^{3}$, $k_{d}^{S}=k_{d}^{NP}=10^{-3}\;s^{-1}$, $\sigma_{S}^{S}=10^{17}\;m^{-2}$, $\sigma_{S}^{NP}=10^{18}\;m^{-2}$, $\rho_{NP}/\rho_{LC}=3.9$, $n_{0}=3\times 10^{19}\;m^{-3}$.

An example of the time dependence of ion-capturing/ion-releasing regimes in liquid crystals doped with contaminated nanoparticles and sandwiched between contaminated substrates is shown in Figure 4.

The type of the observed regime depends on the interplay between the contamination factor of nanoparticles ($\nu_{NP}$) and substrates ($\nu_{S}$), adsorption/desorption parameters ($k_{a}^{NP}$, $k_{d}^{NP}$, $K_{NP}=k_{a}^{NP}/k_{d}^{NP}$, $k_{a}^{S}$, $k_{d}^{S}$, $K_{S}=k_{a}^{S}/k_{d}^{S}$, $\sigma_{S}^{NP}$, $\sigma_{S}^{S}$), the concentration of nanoparticles ($\omega_{NP}$) and their size ($R_{NP}$), initial concentration of ions in liquid crystals ($n_{0}$), and cell thickness (d).

The time dependence of the ion-capturing regime is represented by dashed and short-dotted curves in Figure 4. Dotted and short-dashed curves show the kinetics of the ion-releasing regime (Figure 4). The kinetics of ion-capturing/ion-releasing processes is characterized by two time constants, $\tau_{NP}=1/k_{d}^{NP}\left(K_{NP}n_{NP}A_{NP}\sigma_{S}^{NP}+1\right)$ and τS=1kdS(KSσSSd+1), originated from the presence of nanoparticles and substrates, respectively. An interesting feature is the possibility of both monotonous (Figure 4, dashed and dotted curves) and non-monotonous (Figure 4, short-dotted and short-dashed curves) time dependence n(t).

## 3. Conclusions

Since the liquid crystal cell is a major component of practically any electro-optical device utilizing mesogenic materials, the results presented in this paper have important practical implications. Once the cell is filled with liquid crystals, interactions of ions with alignment films and/or nanodopants through the ion adsorption/ion desorption processes result in a strongly pronounced time dependence of its electrical properties. Electro-optical response of liquid crystals, especially at relatively low frequencies, can be affected by the presence of ions in liquid crystals [2,3,51]. As a result, electro-optical properties of the liquid crystal device can also become time-dependent. The knowledge of this time dependence is very important from both scientific and applied perspectives. Findings presented in this paper provide an important information on the kinetics of ion-capturing/ion-releasing processes in liquid crystal cells utilizing nanomaterials and alignment layers. Very important aspect of the present study is the consideration of the ionic contamination of both nanodopants and alignment films. Too often this factor (ionic contamination) is overlooked in existing literature. The presented model shows possible scenarios of time-dependent ion-capturing/ion-releasing regimes in liquid crystal devices and their dependence on the ionic contamination of both nanodopants and alignment layers. Thus, it can be used for the analysis of existing experimental data and will also guide the design of liquid crystal devices utilizing nanoparticles and alignment films.

Some limitations of the proposed model should also be mentioned. The limits and applicability of this approach have already been discussed [41,42,43]. In addition, this model does not consider the nature and origin of ionic contaminants. Different types of materials (liquid crystals, nanodopants, alignment films) and ionic contaminants can be distinguished by using different values of physical parameters used in the model ($k_{d}^{NP}$, KNP=kaNPkdNP, $\sigma_{S}^{NP}$, $k_{d}^{S}$, KS=kaSkdS, $\sigma_{S}^{S}$). The geometry of the cell and orientation of liquid crystals is fixed thus the afore-mentioned physical parameters characterizing the ion-capturing/ion-releasing processes are considered constant.

To summarize, the kinetics of ion-capturing/ion-releasing processes in liquid crystal cells utilizing contaminated nanoparticles and alignment layers exhibits several non-trivial features (Figure 1, Figure 2, Figure 3 and Figure 4). These features originate from the presence of both nanoparticles and alignment layers and from their ionic contamination. The ionic contamination of nanoparticles and alignment layers governs the type of the regime (Figure 1, Figure 2 and Figure 3) and determines whether the observed time dependence n(t) is monotonous or non-monotonous (Figure 4). This time dependence n(t) is described by time constants $\tau_{NP}$ and $\tau_{S}$ characterizing ion adsorption/ion desorption processes on the surface of nanoparticles and alignment layers, respectively. The time constant $\tau_{NP}$ depends on the ion-nanoparticle related adsorption parameters ($k_{d}^{NP}$, KNP=kaNPkdNP, $\sigma_{S}^{NP}$), concentration of nanoparticles $\omega_{NP}$, and their size $R_{NP}$ (Figure 1 and Figure 2). The time constant $\tau_{S}$ is also a function of the adsorption parameters characterizing the ion adsorption/ion desorption processes on the surface of alignment layers ($k_{d}^{S}$, KS=kaSkdS, $\sigma_{S}^{S}$). In addition, it depends on the thickness of the cell (Figure 3). The obtained results offer an efficient way to control the kinetics of ion-capturing/ion-releasing process in liquid crystal devices by changing the concentration of nanoparticles, their size, cell thickness, and the ionic contamination of both nanoparticles and alignment layers.

## Figures and Tables

**Table 1:** Examples of existing experimental data.

Materials (Liquid Crystals Doped with Nano-Objects)	Physical Parameters	Ref.
Nematic liquid crystals (E44) doped with anatase nanoparticles (TiO_2_)	$K_{NP}=10^{-23}\;m^{3}$, $\sigma_{S}^{NP}=0.8\times 10^{18}\;m^{-2}$, $\nu_{NP}=0.00015$, $n_{0}=3\times 10^{19}\;m^{-3}$, d=11.5±0.5 μm	[16,47]
Nematic liquid crystals (E7) doped with carbon nanotubes	$K_{NP}=7\times 10^{-24}\;m^{3}$, $\sigma_{S}^{NP}=10^{18}\;m^{-2}$, $\nu_{NP}=0.0000095$, $n_{0}=2.5\times 10^{18}\;m^{-3}$, d=11.3 μm	[16,48]
Liquid crystals (8OCB) doped with graphene	$K_{NP}=8\times 10^{-24}\;m^{3}$, $\sigma_{S}^{NP}=0.33\times 10^{18}\;m^{-2}$, $\nu_{NP}=0.0000085$, $n_{0}=2.6\times 10^{18}\;m^{-3}$, d=7.0±0.5 μm	[16,49]
Nematic liquid crystals (E44) doped with ferroelectric nanoparticles (BaTiO_3_)	$K_{NP}=10^{-23}\;m^{3}$, $\sigma_{S}^{NP}=5\times 10^{18}\;m^{-2}$, $\nu_{NP}=0$, $n_{0}=2.44\times 10^{18}\;m^{-3}$, d=11.3±0.5 μm	[14,16]

**Table 2:** Examples of existing experimental data.

Materials (Liquid Crystals/Films)	Physical Parameters	Ref.
Nematic liquid crystals (ZLI-4792) sandwiched between substrates with alignment layers made of SiO*_x_*	$n_{0}=2.4\times 10^{19}\;m^{-3}$, d=5−6 μm, $K_{s}=\frac{k_{a}}{k_{d}}\leq 6.7\times 10^{-22}\;m^{3}$, $\sigma_{S}\geq 10^{16}\;m^{-2}$	[31]
Nematic liquid crystals (ZLI-1132, Merck Corp., Kenilworth, NJ, USA) sandwiched between two substrates with alignment layers (Polyimide AL-1051, JSR Corp, Tokyo, Japan)	$n_{0}=3\times 10^{19}\;m^{-3}$, d=4.9 μm, $k_{a}n_{0}=4\times 10^{-4}\;s^{-1}$, $k_{d}=1.3\times 10^{-3}\;s^{-1}$ $\sigma_{S}=10^{16}\;-10^{18}m^{-2}$	[35]

The computed time dependence n(t) is shown in Figure 3a.

## References

[1] Yang D.-K., Wu S.-T. (2006). Liquid Crystal Devices.

[2] Chigrinov V.G. (1999). Liquid Crystal Devices: Physics and Applications.

[3] Naemura S. (1999). Electrical properties of liquid crystal materials for display applications. Mater. Res. Soc. Symp. Proc..

[4] Geis M.W., Bos P.J., Liberman V., Rothschild M. (2016). Broadband optical switch based on liquid crystal dynamic scattering. Opt. Express.

[5] Serak S.V., Hrozhyk U., Hwang J., Tabiryan N.V., Steeves D., Kimball B.R. (2016). High contrast switching of transmission due to electrohydrodynamic effect in stacked thin systems of liquid crystals. Appl. Opt..

[6] Konshina E.A., Shcherbinin D.P. (2017). Study of dynamic light scattering in nematic liquid crystal and its optical, electrical and switching characteristics. Liq. Cryst..

[7] Garbovskiy Y., Glushchenko I. (2015). Nano-objects and ions in liquid crystals: Ion trapping effect and related phenomena. Crystals.

[8] Wu P.C., Lisetski L.N., Lee W. (2015). Suppressed ionic effect and low-frequency texture transitions in a cholesteric liquid crystal doped with graphene nanoplatelets. Opt. Express.

[9] Mun H.-Y., Park H.-G., Jeong H.-C., Lee J.H., Oh B.Y., Seo D.-S. (2017). Thermal and electro-optical properties of cerium-oxide-doped liquid-crystal devices. Liq. Cryst..

[10] Shcherbinin D.P., Konshina E.A. (2017). Ionic impurities in nematic liquid crystal doped with quantum dots CdSe/ZnS. Liq. Cryst..

[11] Sharma K.P., Malik P., Raina K.K. (2016). Electro-optic, dielectric and optical studies of NiFe_2_O_4_-ferroelectric liquid crystal: A soft magnetoelectric material. Liq. Cryst..

[12] Podgornov F.V., Wipf R., Stühn B., Ryzhkova A.V., Haase W. (2016). Low-frequency relaxation modes in ferroelectric liquid crystal/gold nanoparticle dispersion: Impact of nanoparticle shape. Liq. Cryst..

[13] Garbovskiy Y., Glushchenko I. (2015). Ion trapping by means of ferroelectric nanoparticles, and the quantification of this process in liquid crystals. Appl. Phys. Lett..

[14] Hsiao Y.G., Huang S.M., Yeh E.R., Lee W. (2016). Temperature-dependent electrical and dielectric properties of nematic liquid crystals doped with ferroelectric particles. Displays.

[15] Garbovskiy Y. (2016). Switching between purification and contamination regimes governed by the ionic purity of nanoparticles dispersed in liquid crystals. Appl. Phys. Lett..

[16] Garbovskiy Y. (2016). Electrical properties of liquid crystal nano-colloids analysed from perspectives of the ionic purity of nano-dopants. Liq. Cryst..

[17] Tomylko S., Yaroshchuk O., Kovalchuk O., Maschke U., Yamaguchi R. (2012). Dielectric properties of nematic liquid crystal modified with diamond nanoparticles. Ukrainian J. Phys..

[18] Samoilov A.N., Minenko S.S., Fedoryako A.P., Lisetski L.N., Lebovka N.I., Soskin M.S. (2014). Multi-walled vs. single-walled carbon nanotube dispersions in nematic liquid crystals: Comparative studies of optical transmission and dielectric properties. Funct. Mater..

[19] Yadav S.P., Manohar R., Singh S. (2015). Effect of TiO_2_ nanoparticles dispersion on ionic behaviour in nematic liquid crystal. Liq. Cryst..

[20] Garbovskiy Y. (2016). Impact of contaminated nanoparticles on the non-monotonous change in the concentration of mobile ions in liquid crystals. Liq. Cryst..

[21] Garbovskiy Y. (2016). Adsorption of ions onto nanosolids dispersed in liquid crystals: Towards understanding the ion trapping effect in nanocolloids. Chem. Phys. Lett..

[22] Urbanski M., Lagerwall J.P.F. (2017). Why organically functionalized nanoparticles increase the electrical conductivity of nematic liquid crystal dispersions. J. Mater. Chem. C.

[23] Garbovskiy Y. (2017). Nanoparticle enabled thermal control of ions in liquid crystals. Liq. Cryst..

[24] Garbovskiy Y. (2017). Ions in liquid crystals doped with nanoparticles: Conventional and counterintuitive temperature effects. Liq. Cryst..

[25] Liu H., Lee W. (2010). Time-varying ionic properties of a liquid-crystal cell. Appl. Phys. Lett..

[26] Wu P.-C., Yang S.-Y., Lee W. (2016). Recovery of UV-degraded electrical properties of nematic liquid crystals doped with TiO_2_ nanoparticles. J. Mol. Liq..

[27] Takatoh K., Hasegawa M., Koden M., Iton N., Hasegawa R., Sakamoto M. (2005). Alignment Technologies and Applications of Liquid Crystal Devices.

[28] Furuichi K., Xu J., Furuta H., Kobayashi S., Yoshida N., Tounai A., Tanaka Y. (2002). 38.4: Effect of Ion Capturing Films on the EO Characteristics of Polymer-Stabilized V-FLCD. SID Symp. Dig. Tech. Pap..

[29] Furuichi K., Xu J., Inoue M., Furuta H., Yoshida N., Tounai A., Tanaka Y., Mochizuki A., Kobayashi S. (2003). Effect of Ion Trapping Films on the Electrooptic Characteristics of Polymer-Stabilized Ferroelectric Liquid Crystal Display Exhibiting V-Shaped Switching. Jpn. J. Appl. Phys..

[30] Kobayashi S., Xu J., Furuta H., Murakami Y., Kawamoto S., Ohkouchi M., Hasebe H., Takatsu H. (2004). Fabrication and electro-optic characteristics of polymer-stabilized V-mode ferroelectric liquid crystal display and intrinsic H-V-mode ferroelectric liquid crystal displays: Their application to field sequential full colour active matrix liquid crystal displays. Opt. Eng..

[31] Huang Y., Bos P.J., Bhowmik A. (2010). The ion capturing effect of 5 SiO*_x_* alignment films in liquid crystal devices. J. Appl. Phys..

[32] Huang Y., Bhowmik A., Bos P.J. (2012). Characterization of Ionic Impurities Adsorbed onto a 5° SiO*_x_* Alignment Film. Jpn. J. Appl. Phys..

[33] Huang Y., Bhowmik A., Bos P.J. (2012). The effect of salt on ion adsorption on a SiO*_x_* alignment film and reduced conductivity of a liquid crystal host. J. Appl. Phys..

[34] Basu R., Lee A. (2017). Ion trapping by the graphene electrode in a graphene-ITO hybrid liquid crystal cell. Appl. Phys. Lett..

[35] Mizusaki M., Miyashita T., Uchida T., Yamada Y., Ishii Y., Mizushima S. (2007). Generation mechanism of residual direct current voltage in a liquid crystal display and its evaluation parameters related to liquid crystal and alignment layer materials. J. Appl. Phys..

[36] Mizusaki M., Miyashita T., Uchida T. (2010). Behavior of ion affecting image sticking on liquid crystal displays under application of direct current voltage. J. Appl. Phys..

[37] Mizusaki M., Miyashita T., Uchida T. (2012). Kinetic analysis of image sticking with adsorption and desorption of ions to a surface of an alignment layer. J. Appl. Phys..

[38] Mizusaki M., Yoshimura Y., Yamada Y., Okamoto K. (2012). Analysis of ion behavior affecting voltage holding property of liquid crystal displays. Jpn. J. Appl. Phys..

[39] Xu D., Peng F., Chen H., Yuan J., Wu S.-T., Li M.-C., Lee S.-L., Tsai W.-C. (2014). Image sticking in liquid crystal displays with lateral electric fields. J. Appl. Phys..

[40] Barbero G., Evangelista L.R. (2006). Adsorption Phenomena and Anchoring Energy in Nematic Liquid Crystals.

[41] Garbovskiy Y. (2016). Adsorption/desorption of ions in liquid crystal nano-colloids: The applicability of the Langmuir isotherm, impact of high electric fields, and effects of the nanoparticle’s size. Liq. Cryst..

[42] Garbovskiy Y. (2016). The purification and contamination of liquid crystals by means of nanoparticles. The case of weakly ionized species. Chem. Phys. Lett..

[43] Garbovskiy Y. (2017). Ions and size effects in nanoparticle/liquid crystal colloids sandwiched between two substrates. The case of two types of fully ionized species. Chem. Phys. Lett..

[44] Riley K.F., Hobson M.P., Bence S.J. (1997). Mathematical Methods for Physics and Engineering.

[45] Marczewski A.W. (2010). Analysis of kinetic Langmuir model. Part I: Integrated kinetic Langmuir equation (IKL): A new complete analytical solution of the Langmuir rate equation. Langmuir.

[46] Gonen Y., Rytwo G. (2007). A full analytical solution for the sorption–desorption kinetic process related to Langmuir equilibrium conditions. J. Phys. Chem. C.

[47] Tang C.Y., Huang S.M., Lee W. (2011). Electrical properties of nematic liquid crystals doped with anatase TiO_2_ nanoparticles. J. Phys. D Appl. Phys..

[48] Jian B.R., Tang C.Y., Lee W. (2011). Temperature-dependent electrical properties of dilute suspensions of carbon nanotubes in nematic liquid crystals. Carbon.

[49] Wu P.W., Lee W. (2013). Phase and dielectric behaviors of a polymorphic liquid crystal doped with graphene nanoplatelets. Appl. Phys. Lett..

[50] Garbovskiy Y. (2017). Ion capturing/ion releasing films and nanoparticles in liquid crystal devices. Appl. Phys. Lett..

[51] Ciuchi F., Mazzulla A., Pane A., Adrian Reyes J. (2007). ac and dc electro-optical response of planar aligned liquid crystal cells. Appl. Phys. Lett..

